# Supplementary material for: Microbiological profile of diabetic foot ulcers in Kuwait
Source: PLoS One. 2020 Dec 30;15(12):e0244306. doi: 10.1371/journal.pone.0244306 (PMC7773204; doi:10.1371/journal.pone.0244306)
Supplement: Attachment — Submitted filename: Response to Reviewers.docx [file pone.0244306.s002.docx]

**Response to Reviewers**

**Reviewer #1:**

**While the topic of the manuscript is indeed interesting and relevant, I have a set of concerns/queries, as outlined below:**

We would like first, to thank the reviewer for the overall positive feedback on our manuscript. We also thank him/her for the valuable and detailed suggestions made to make our findings more focused and to enhance the quality of our draft.

In our reply, we have addressed all the queries that were raised and changes, whenever applicable, were incorporated in the revised manuscript and shown in blue color. Please find below our point-by point responses to the reviewer’ comments.

- The first cited reference is a previous study published by part of the authors also involved in the current manuscript: “Messenger G, Masoetsa R, Hussain I, Devarajan S, Jahromi M: Diabetic foot ulcer outcomes from a podiatry led tertiary service in Kuwait. Diabete Foot Ankle 2018, 9:1471927.” Much of the same methodology was used, but the first paper looked at different outcomes, in the time span 2014-2016. The following microbiology data is reported in this previous article: “Microbiology samples were collected from 150 DFUs, of which nearly 40% were positive for bacterial growth. The most common microorganisms were Gram-negatives (16%), and 11 (3%) new cases of Methicillin-resistant Staphylococcus aureus (MRSA) were identified.” I find it hard to understand why a new paper would be needed, and why publish these two analyses as two separate manuscripts.

We thank the reviewer for this valuable comment and his/her detailed feedback on potential overlap between our current study with the previous one which was recently published by our institute.

Indeed, in our previous study the main objective was to evaluate the incidence, risk factors and treatment outcomes of a podiatry led, evidence-based diabetic foot ulcer (DFU) clinic for patients referred to our institute during the period from October 2014 till December 2016. As outcomes, our previous study allowed us to estimate the incidence of diabetic foot ulceration, days to heal, healing rate and the risk factors influencing healing which were in accordance with other multidisciplinary facilities with podiatry input.

Nevertheless, in our current study, we focused on the diabetic foot infection (DFI) and its associated microbiological profile. We have also taken into consideration the distribution of microbial isolates according to, sex, age, diabetes control and for the first-time wound grade. Furthermore, in our current study we covered a longer period of time (2011-2017) and included more patients (513) categorized by age, gender and diabetes control. In the present study, we have also analyzed separately the monomicrobial and polymicrobial wound infections and assessed their correlations with other risk factors. Despite the population study was larger and different, the clinically infection rate for DFU was comparable (49.3% in the current study versus 40% in the previous study from other healthcare providers). Finally, in our previous publication, we did not report any details about the microorganisms infecting DFU except of the rate of gram-negative and MRSA as mentioned by the reviewer.

- Line 24: The phrasing should be revised, since it is an overstatement to say that the study’s aim was to characterize DFU “within Kuwait”. The same can be said for the conclusions on line 40 “patterns in Kuwait”, and for the manuscript’s title. This data reflects, at best, the population attending the DDI center in Kuwait in the study time span.

We thank the reviewer for these key comments. In fact, the diabetic patients attending Dasman Diabetes Institute (DDI) were referred from all governorates in Kuwait and to some extend represent all Kuwait population. Nevertheless, and as suggested by the reviewer, in the revised manuscript we have changed the statement in Line 24 and Line 40 as the following:

*“The aim of our study was to determine the microbiological profile of infected ulcers in patients attending Dasman Diabetes Institute (DDI) clinics in Kuwait and to analyze the distribution of microbial isolates according to wound grade, gender, age and diabetes control.”*

*“****Conclusion:*** *Our results provide an updated picture of the DFI patterns in patients attending Dasman Diabetes Institute (DDI) clinics in Kuwait and support the observed regional variability of DFI pathogens, while highlighting the need for the early and appropriate treatment of infected foot ulcers to enhance wound healing and improve clinical outcomes.”*

- Furthermore, this study population attending the DDI center should be clearly defined on line 93, where a brief description of the hospital is first given.

As suggested by the reviewer, we have added the following statement on line 93 and now it reads:

*“DDI is a specialized outpatient center helping patients with diabetes mellitus manage their blood glucose levels and treat diabetes complications. Diabetic patients with complications were referred to DDI from all governorates in Kuwait and might be considered representative of all Kuwait population. During this period, 835 patients with DFU were referred to the Podiatry Department, this represented around 120 patients per year.”*

- Line 26: A brief statement regarding the criteria used for selecting patients for the study should be included here. For example: all consecutive patients attending the Institute within a specific time span.

As suggested by the reviewer and for further clarity of the method section, we have added the following statement on line 26:

***“Methods:*** *We collected and analyzed clinical data and samples from 513 diabetic patients with foot ulcers referred to our podiatry clinic at DDI from Jan 2011 till Dec 2017.”*

- Lines 38 and 39: It would be easier to follow the numbers if the same order is used for reporting. In these phrases female cases are reported first and then male cases first.

We totally agree with the reviewer about this comment. Accordingly, we have changed the order as suggested:

*“Finally, a higher percentage of women has controlled HbA1c levels (19.41% versus 11.95% in men) and more women in this group displayed non-infected wounds (60.6% and 43.90% for women and men, respectively).”*

- Line 41: “the observed regional variability of DFI pathogens” – this study has not looked at regional variability specifically, only the discussion section provides a comparison with different regions/countries. However, this comparison cannot truly reflect a regional variability, since differences in methodology can be reasonably expected. Even we are using international and National standardized methodology in Identifying and processing the samples?

We agree with the reviewer that we have not made a comparative study to assess the regional variability. Our current study covering a segment of Kuwaiti patients does, however, support this regional variability as we have detailed in our discussion section. We also agree that different studies have used different methodologies and thus comparing them may not very accurate.

Accordingly, we have removed this statement from the Abstract.

- Line 42: Since this was not a study of wound healing time/quality, or of clinical outcomes, the following phrase is not a direct conclusion of the study: “to enhance wound healing and improve clinical outcomes.”

As suggested by the reviewer, we have changed our statement and now it reads as:

“Our results provide an updated picture of the DFI patterns and antibiotics resistance in patients attending Dasman Diabetes Institute (DDI) clinics in Kuwait which might help in adopting the appropriate treatment of infected foot and improving clinical outcomes.”

- Line 49: “The lifetime risk of developing a DFU is estimated to be 15 to 25% [1, 2].” This phrase requires clarification. Are the findings from literature reported here valid only for patients with diabetes? Or for the general population? Furthermore, it is not clear from which literature reference this information was derived, since the reference quoted here (#1) cites the following different information, for the same reported percentages, and further citing another literature sources: “Diabetic foot ulcers (DFUs) continue to be a leading cause of non-traumatic lower limb amputation [1], with an estimated lifetime risk of 12–25% [2–4].” Citations should generally point towards the initial source and it should be checked that the intended meaning is portrayed.

We thank the reviewer for this valuable comment and apologize for this confusion in citing the references. Accordingly, we have made the necessary modifications and introduced the original references as following:

- *Reiber GE, Smith DG, Carter J, et al. A comparison of diabetic foot ulcer patients managed in VHA andnon-VHA settings. J Rehabil Res Dev. 2001;38 (3):309–317.*
- *Ramsey SD, Newton K, Blough D, McCulloch DK, Sandhu N, Reiber GE, Wagner EH. Incidence, outcomes, and cost of foot ulcers in patients with diabetes. Diabetes Care 1999;22:382–7. 1992;152:610–6.*
- *Lavery LA, Armstrong DG, Wunderlich RP, Tredwell J, Boulton AJ. Diabetic foot syndrome: evaluating the prevalence and incidence of foot pathology in Mexican Americans and non-Hispanic whites from a diabetes disease management cohort. Diabetes Care. 2003;26:1435-1438.*

- Line 53: “Despite the fact that the prevalence of diabetes in Kuwait is ranked among the highest worldwide” – how high?

As suggested by the reviewer, we added more details on the diabetes status in Kuwait as following:

*“**Despite the fact that the prevalence of diabetes in Kuwait (22.0%) is ranked among the top 20 highest worldwide [5] IDF 2019”*

- Line 64: Please recheck italicizing and capitalization. All bacterial genus and species names should be written in italic font, whereas “spp.” or “species” should not. Genus names should be capitalized, while species names should not.

We have checked and corrected all the names throughout all the manuscript as requested by the reviewer.

- Lines 72-73: Depth of the wound and sampling technique can also be involved.

We agree with the reviewer on this extra source of variability and we added them to the text as suggested. Now the text reads as following:

*“These discrepancies could be related to various factors, such as the presence of different causative organisms at different times, geographical variations and ecologies, socio-economic conditions, hygiene, accessibility to effective health care services, the type and the severity of the infections, or depth of the wound and sampling technique [12, 22].”*

- Line 74: The term “colonizers” should be used only when talking about colonization, i.e., not infection.

As suggested the word “colonizers” was substituted with “pathogens” in this context and it was also checked throughout the manuscript.

- Line 100 and ethics statement: The Declaration of Helsinki generally refers to the rights of subjects in interventional clinical trials. Also, a statement should be included regarding the waiver of informed consent by an ethics committee.

As requested by the reviewer, this statement was rephrased with the appropriate terms as following:

*“Approval and a waiver from the need to provide written informed consent were obtained from The Ethical Review Board of DDI for the access and use of anonymized data from the LIS for the purpose of publication.”*

- Line 128: CAP should be defined at the first use in the text.

As requested, CAP was defined in the text.

- Line 149: Check spelling and italicization: Acinitobacter, etc.

As requested, spelling and italicization of names were checked and corrected whenever need throughout all the manuscript.

- Line 158: Check producer for this SPSS version. It should be: IBM Corp., Armonk, NY, USA)’.

As requested by the reviewer, the details have checked and corrected:

*“We analyzed the data using the statistical SPSS software, v22.0 (IBM SPSS Statistics for Windows, IBM Corp. Armonk, NY, USA). “*

- Line 159: Student’s t test is only appropriate for variables with parametric distribution. Was variable distribution checked for all continuous variables?

We thank the reviewer for this comment. Indeed, and as mentioned in our manuscript, we evaluated differences between continuous and categorical variables using two appropriate statistical tests; the Student’s t-test for the continuous variables with parametric distribution and the Chi-square test for the non-parametric categorical variables.

- Lines 205, 232, 308 and other occurrences: By Staphylococcus aureus, are the authors referring to MSSA?

Yes, we are referring to Methicillin Sensitive Staphylococcus aureus (MSSA). We have added it at the first occurrence in the text of our revised version for further clarity.

The text now reads as:

*“Interestingly, 47.7% of grade A and 42.9% of grade C DFUs grew at least one of the following bacteria, Methicillin Sensitive Staphylococcus aureus (MSSA), MRSA, and Pseudomonas aeruginosa (data not shown).”*

- Lines 226-229: Check the use of italic font.

We have checked and corrected all the italicization and capitalization of strain names throughout all the manuscript as requested by the reviewer.

- Line 243: Correlation analysis is only applicable for continuous variables. An “association” should be discussed instead.

We thank the reviewer for this valuable comment. Indeed, we have checked the text and we have substituted the word “correlation” with “association” when appropriate.

- Lines 245 and all antimicrobial names: Check capitalization.

We have checked and corrected all the capitalization of strain and antimicrobial names throughout all the manuscript as requested by the reviewer.

- Line 248: The 97.5% susceptibility to linezolid should be discussed as this is an important, worrisome finding.

As requested by the reviewer, the susceptibility of Staphylococcus *aureus* to linezolid was discussed ad the following statement was added to the discussion section as follow:

*“However, we cannot rule out other potential reasons affecting this rate, such as the younger age of our studied population. It is noteworthy that in our study 2.5% of Staphylococcus aureus isolates have shown resistance to Linezolid. Despite most of Staphylococcus aureus isolates including MRSA, are susceptible to linezolid, the emergence of linezolid-resistant MRSA has been, however, previously reported [45, 46].”*

- Line 252: The decreased susceptibility to carbapenems should be discussed.

As suggested by the reviewer, the following statement was added to the discussion section in our revised manuscript:

“While *E. coli* and *Klebsiella species* were 100% sensitive to Carbapenems, *Pseudomonas aeruginosa* was relatively less sensitive to this class of antibiotics (88.4% and 85% sensitivity, respectively to Meropenem and Imipenem). These results are comparable to a previous report from Kuwait educational hospital where *Pseudomonas aeruginosa was* 10% resistance to Imipenem [15].”

- Figure 1: “Controlled male/female” and “Uncontrolled male/female” sounds a bit awkward and should be revised. Furthermore, a figure legend should clarify that this term refers to glycemic control and the values for control should be stated here, i.e., 6.5.

As suggested by the reviewer, the confusing group titles in the figure were changed and a separate figure legend was added on page 21 in the revised version of our manuscript.

- Full statistical comparisons should be included in the relevant tables and only discussed in the text.

Statistical comparison was added appropriately to the Tables as requested by the reviewer.

- Table 1 is hard to follow.

We agree with the author for this point. To further simplify Table 1, we have added a title to one column and 3 footnotes after the table to explain what these percentages are. However, we did not include full statistical comparison in this table to keep it easy for reading as only the mean age of males and females was statistically significantly different. This significance was already labeled with a star and a footnote in this table.

- Table 2, Table S1 and analysis in the manuscript text: Please revise the definition of “controlled” and “uncontrolled”. At the current moment a patient with 6.5 does not fit into any of these categories. Please revise and recalculate.

We thank the reviewer for this important comment, and we apologize for this confusion. Indeed, there was a mistake in the Tables and some sections in the text where sometimes we used “>6.5%” and other places we used “≥6.5%”for HbA1c values equal or greater than 6.5% by using the comparative “>” instead of “≥”. Thus, the HbA1c value equal to 6.5% seemed to not be included. Accordingly, we have well defined the controlled and uncontrolled HbA1c values at the first use in the results section on page 6. We have also fixed this comparison symbol in the tables of the revised version of our manuscript. Nevertheless, it does not induce any changes in the results as we have already taken into consideration the patients with HbA1c=6.5% as part of the uncontrolled diabetes group in our data analyses.

- Table S1: This table is not very relevant. Furthermore, in the A3 rows, how can a “controlled” patient have an HBa1c level > 10?

As suggested by the reviewer, this Table was removed, and the related data was briefly mentioned in the text.

- Table 3 is not needed, as much of this information is already presented in the text.

As suggested by the reviewer, this Table was removed, and the related data was briefly mentioned in the text.

- Table 4: Please revise the use of italic font and spelling of some of the bacterial names.

We have checked and corrected all the spelling, italicization, and capitalization of strain names throughout all the manuscript as requested by the reviewer.

- Table 5 is a bit hard to follow because of the different column names in the first and second half of the table.

As suggested by the reviewer, we have simplified the Table and split it to 2 sub-tables as the headings are different between gram-positive and gram-negative strains.

- Table 5: Data on cefoxitin screening for S. aureus should also be included.

In our study, we used cefoxitin only with the panels of the Microscan and not cifotoxin disks to distinguish between MRSA which are all resistant to cefoxitin in opposition to *S. aureus* which are all sensitive to cefoxitin.

- The references used for comparison in the Discussion section should overall be read more carefully, to avoid misinterpretation of the data when citing their results.

As suggested by the reviewer, we have checked the used references for both pertinence and reduction of the number of references.

- Line 271: These are not “the first estimates”, as the authors have already published data on this topic, as mentioned above.

This statement was removed as suggested.

- Lines 286-289: When discussing mono- vs poly-microbial infection, it would be important to look at the sampling and processing techniques in each study, otherwise the overall numbers cannot be compared.

We agree with the reviewer on this point. Accordingly, we have removed the references (36, 37 and 38 in the initial version) and we have changed this statement as following:

*“Globally, the number of isolates found in DFIs varies widely. Polymicrobial infection rates have been found to be more prevalent in studies from a number of different countries including 83.7% in Portugal [32], 60% in South India [21], 55.7% in Mumbai, India [33], and 40% in Italy [34], whereas others have reported higher rates of monomicrobial infections [35-37]. Nevertheless, this variation in prevalence could be also related to sampling and processing techniques in each study.”*

- Line 340: It is not clear what this phrase refers to: “the most persistent pathogens in healthcare facilities worldwide”.

In this phrase, we meant *Staphylococcus aureus*. To make this statement clear, we have rephrased the sentence as following:

*“… which also reported a high frequency of Staphylococcus aureus in DFI. This latter strain causes the majority of human infections and has proven to be among the most persistent pathogens in healthcare facilities worldwide [42].”*

- Lines 341-344: The authors state that: “The presence of MRSA in our study was only 5.0% of the total isolates detected. This rate is much lower than European statistics. Recently, The European Centre for Disease Prevention and Control (ECDC) reported that the prevalence of MRSA decreased across Europe from 19.6% in 2014 to 16.9% in 2017 [43].” However, these numbers cannot be compared, since the cited report reflects the percentage of MRSA isolates from the total number of S. aureus isolates, while the percentage reported in the current manuscript does not.

We thank the reviewer for this valuable comment. Indeed, the numbers are not directly comparable as they are now. Thus, we have recalculated the percentage of MRSA isolates from the total number of *S. aureus* and which is now 21%. Consequently, we have removed all this statement from our revised manuscript in relation with the differences in MRSA. The text now reads as:

*“This latter strain causes the majority of human infections and has proven to be among the most persistent pathogens in healthcare facilities worldwide [42]. The MRSA rate in study is also comparable to the range reported in other countries, although prevalence rates vary widely [43, 44]. The reduction in polymicrobial infections in our study…”*

- Line 345: Reduction compared to what?

- Line 346: Improvement compared to what/when?

Those comparison were referring to previous reports from Kuwait. We have thus added the following statement to make the sentence more accurate.

*“The reduction in polymicrobial infections in our study, in comparison to previous reports from Kuwait [13, 15], may reflect an improvement of healthcare system and in patients’ hygiene. However, we cannot rule out other potential reasons affecting this rate, such as the younger age of our studied population.”*

- Line 354: What differences in healthcare practice?

In this statement we meant the sample collection and processing aside with standards used by different healthcare system which might vary from a region to another. To make this statement more precise, we have changed the sentence as following:

*“These observations on the prevalence of Pseudomonas aeruginosa suggest regional variability probably due to microbial environments and healthcare practices such as sample collection and processing.”*

- The reference list is quite long for an original article, and many of the references included here are either outdated, or not directly relevant to the current study.

As suggested by the reviewer, the list of used references has been revised and the less relevant ones were removed throughout the text.

- Data availability statement: “All generated data and resources are reported in this manuscript and there is no other data to be provided.” The source dataset should also be made available.

As suggested by the reviewer, this statement has been amended in our revised version.

- English language is overall ok, but it should be revised to address occasional mistakes or errors.

As mentioned in the Acknowledgement section, the English language of our manuscript has been edited by a professional company (Enago). For our revised version, our co-author Grace Messenger has checked the text again for any mistakes.

**Reviewer #2:**

I **would like to thank the authors for their paper. The topic of diabetic foot ulcers and the microbiological profiles will be of great interest to readers. While the paper is well-written, I believe there is a major methodological error in this study, which inhibits the ability to draw valid conclusions. Please refer below to my full list of thoughts, comments, suggestions, and questions:**

We would like first, to thank the reviewer for the overall positive feedback on our manuscript. We also thank him/her for the valuable suggestions made to enhance the quality of our draft.

In our reply, we have addressed all the queries that were raised and changes, whenever applicable, were incorporated in the revised manuscript and shown in blue color. Please find below our point-by point responses to the reviewer’ comments.

• Lines 148-156: Materials and Methods: Antimicrobial Susceptibility Testing- Please include the antibiotic list and concentrations used. Adding the concentrations of discs used will ensure reproducibility.

As requested by the reviewer, the list and concentrations of used antibiotics have been added to the Materials and Methods section as following:

*“Susceptibilities to selected antibiotics for the common isolated bacteria (Staphylococcus aureus, Pseudomonas aeruginosa, Klebsiella pneumoniae, Escherichia coli, Acinetobacter sp and Enterobacter sp) were examined. Antimicrobial sensitivity testing of all isolates was performed on diagnostic sensitivity test plates according to the Kirby-Bauer method [29] following the definition of the Clinical and Laboratory Standards Institute (CLSI, 2014). Antibiotics and their concentrations used for antimicrobial susceptibility testing, are as following: Amikacin 30ug, Ampicillin10ug, Co-Amoxi/clav30 ug , Ceftazidime 30ug , Ciprofloxacin 5ug, Clindamycin 2ug, Ceftriaxone 30ug, Cefotaxime 30ug, Erythromycin 15ug, Gentamicin 10ug, Imipenem 10ug, Linezolid 30ug, Meropenem 10ug, Piperacillin/Tazobactam 110ug, Trimethoprim-Sulfamethoxazole 25ug, and Vancomycin 30ug. Bacterial inoculums were prepared by suspending the freshly grown bacteria in 5mL sterile saline solution. A sterile cotton swab was used to streak the surface of Mueller Hinton agar plates (Oxoid, Basingstoke, UK). Filter paper disks containing a designated concentration of the antimicrobial drugs obtained from Becton and Dickinson Company (Franklin Lakes, NJ) were used.”*

• Line 208: Results: Microbial Isolate Etiologies in DFUs with clinical infection- The authors indicate that to characterize the microbiological profile they only analyzed “clinically infected wounds”. The “clinically uninfected” could essentially have served as the control group. When characterizing microbiological profiles, it is very essential to have a control group that would help understand the “baseline microbiological composition”, and thus would help make valid conclusions about what truly is the microbiological composition of the diseased state (in this case clinically infected samples). Not characterizing the microbiological composition of a control is a major methodological error of this study.

We totally agree with the reviewer on this point and the added value of using the clinically uninfected wounds as controls. Nevertheless, and following long discussions with expert colleagues in the fields, they recommended to focus our paper on the clinically infected wounds to avoid confusion with colonized but non-infected wounds despite we did collect the microbial profile for all patients.

• Line 244: Results: Antimicrobial susceptibility pattern- Please switch the outcome to % resistant instead of %sensitive/susceptible. Since the authors report % resistance for Acinetobacter and Enterobacter species, I believe it would be beneficial if the authors continue to report the results of the antimicrobial resistance patterns as % resistant.

As suggested by the reviewer, the whole paragraph was changed to resistance instead of sensitivity for the antibiotics. Now it reads as follow:

***“****The most prevalent gram-negative and gram-positive pathogens isolated from our DFI patients were tested for their sensitivity against a wide range of antibiotics and results are shown in Table 5. Staphylococcus aureus showed low resistance to vancomycin and linezolid (0% and 2.5%, respectively) but was more resistant to other tested antibiotics (53.6% and 83% for erythromycin and ampicillin, respectively). Of the gram-negative isolates, Pseudomonas aeruginosa was relatively less resistant to meropenem, amikacin, imipenem, gentamicin, and ciprofloxacin (11.6%, 13%, 15%, 20% and 22.8%, respectively). Meanwhile, Escherichia coli strains were not resistant or showing very low resistance to imipenem, meropenem, amikacin, piperacillin/ tazobactam and gentamicin (0%, 0%, 0%, 0% and 2.7%, respectively). Klebsiella species were not or barely resistant to imipenem, meropenem, piperacillin/tazobactam, and gentamicin (0%, 0%,7% and 9%, respectively). Finally, Acinetobacter and Enterobacter species were resistant to ceftriaxone (66.7−68.2%) while being moderately to highly sensitive to the other antibiotics (Table5).”*

• Discussion: Please include the significance of the antimicrobial resistance testing results in the discussion and what this would mean for the clinical management of DFUs and DFIs.

We thank the reviewer for this valuable comment. In our revised version, we have added some sentences here and there in the discussion section about the significance of antimicrobial resistance as requested.

• Discussion: I would also recommend the authors to include another limitation of the study in their discussion. Since the authors have employed a culture-based approach to characterize microorganisms, some microorganisms might be unculturable. Using whole genome sequencing (WGS) to characterize microbiological profiles may provide a more accurate representation of microorganism profiles. Not only will WGS characterize non-culturable organisms, but the number of reads can be used to estimate the “load” of microorganisms in DFUs.

We totally agree with the reviewer on this limitation in our study. Unfortunately, we did not use the WGS as we did not have access to such technology during our study. Accordingly, and as suggested by the reviewer, we have added this limitation in our study as following:

*“Despite the relatively large number of patients and the long period of time covered in our study, some limitations need to be highlighted. One unavoidable pitfall is the fact that our samples are a mixture of swabs and tissue samples that may not give consistent results, in particular with regards to aerobic and anaerobic pathogens as we did not include any condition or analysis of the latter group. In this context, using whole genome sequencing (WGS) approach may provide a more accurate representation of microorganism profiles including non-culturable organisms and a better estimate of the microorganism “load” in DFUs. Furthermore, in such studies, only DFU with clinical infection are usually investigated at the microbiological level whereas here we initially included both DFU with and without clinical infection.”*

• Line 557 Table S1: I would recommend the authors label each stage and grade and depth for clarity. For instance, label A0 with “Pre- or post-ulcerative lesion completely epithelialized”, A1 with “Superficial wound not involving tendon, capsule or bone” so on and so forth.

As per request from Reviewer 1, Table S1 has been removed from our revised version.

**Reviewers' comments:**

**Reviewer #3:**

**Microbiological profile of diabetic foot ulcers in Kuwait**

This paper describes a retrospective study of the microbiology profile diabetic foot ulcers in Kuwait. It adds little if any novel findings to the field; however, such is the nature of studies of this type and that should not preclude publication.

Overall, the paper is well written and well-presented. However, there are several major and minor revisions which we need to be addressed before publication.

We would like first, to thank the reviewer for the overall positive feedback on our manuscript. We also thank him/her for the valuable suggestions made to enhance the quality of our draft.

In our reply, we have addressed all the queries that were raised and changes, whenever applicable, were incorporated in the revised manuscript and shown in blue color. Please find below our point-by point responses to the reviewer’ comments.

**Major revisions**

1. It would be useful if the authors could present a breakdown of the number of patients whose ulcers (x/260) or infections (x/253) were investigated using either swab or tissue sample collection. The authors are correct in their discussion when they highlight that this is a limitation of this work. It may also reflect the high proportion of coagulase-negative *staphylococcus* identified in table 4, and the potential for contaminating skin flora to be detected by superficial swabs should be highlighted in the discussion.

We thank the reviewer for this valuable comment. As suggested, we have added a supplementary Table (Table S1 in the revised version of our manuscript) with details of samples collected. We have further combined sample sources with the microorganism isolated from those samples. This combines and satisfies both suggestions from the reviewer (point 1. And point 2.)

2. This paper should include a clear breakdown of the number of bacterial isolates detected from the 253 infected patients, regardless of their Texas grade, and the frequency with which each bacterial species was identified. This should come in table format**.**

We agree with the reviewer on this point and the table was added as mentioned in the previous point. (Table S1)

**Minor revisions**

1. There are multiple instances in this paper where the abbreviation for diabetic foot infection or diabetic foot ulcer is used incorrectly. The authors need to carefully check that every time they have used a three-letter acronym (DFU vs. DFI) they have used the correct one. Examples of lines where attention is required include:

31-‘DFU infections’

80-‘microbes isolated from DFIs from a different DFUs using the University of Texas…’ This needs clarifying, I think you mean provide power microbes isolated from DFI’s from different ulcer grades.

82-should be DFI pathogens

83-should be the microbial profile of DFIs

112-

211-an infected DFU is a DFI

These examples are not exhaustive, there may be others.

We Thank the reviewer for this comment. As suggested, we have checked and corrected, in all the manuscript, the use of these 3-letter acronyms and their pertinence.

2. Throughout the manuscript you have used sex and gender interchangeably. Please use sex throughout.

As suggested by the reviewer, all the words “gender” were substituted with the word “sex” throughout the manuscript.

3. Please define the abbreviation CAP-line 128

The abbreviation CAP was defined as requested:

*“College of American Pathologists (CAP)”*

4. There is a formatting issue on lines 192 and 201-please correct.

Also on these lines, please insert ‘df’ if you refer to degrees of freedom in the parentheses. At it’s first use degrees of freedom should be defined as ‘df’.

This was the symbol Chi ((ꭓ2) but during the conversion of the manuscript from Word text to PDF it was not properly converted. We checked that in the final Word version of our revised manuscript is correctly spelled.

5. Line 193-please write ‘years’ in full

Corrected as requested

6. Line 202. If you have used an ‘F test’ please also include this in your methods.

Indeed, we used the F test and now it is mentioned in the statistical section of the methods.

7. Line 209-please remind the reader of the number of clinic infected wounds to which you are referring – e.g. (n = x)

As suggested by the reviewer, the number of patients with clinical infection was added and now the text reads as”

*“To better characterize the microbial etiology related to DFI, in the remaining part of our study and analyses we only included the clinically infected wounds (n=253).”*

8. Throughout the manuscript you have been inconsistent in your spacing of figures. For example, n=x vs. n = x. Please choose one format and stick with it throughout.

Checked and corrected as requested by the reviewer.

9. Line 248 please insert ‘was’ between ‘but less’

The sentence has been now changed following the request of another reviewer and the word “was” has been inserted as requested.

10. Line 272, should read ‘contained twice as many men as women’

The sentence has been changed as requested.

*“Our study population contained twice as many men as women with DFI, yet, our clinics have equal access to both sexes.”*

11. Line 290, the claim that most diabetic foot infections are ‘dominated’ by Gram-negative strains is based on five references here. All these references come from papers published in India or China. You cannot make a statement about ‘most reports’ easier handfuls of papers from very limited geographical sources. As it happens, certainly in Western Europe and the United States, it is most often gram-positive cocci which predominate in these infections. I suggest removing this sentence.

We agree with reviewer on this point and the sentence has been removed as requested.

12. Line 305, extra space has been inserted

The extra space has been removed as requested.

13. line 339, you mean species not strain.

Yes, we mean species. We have subtitled it as requested

14. Table 1, please sort out formatting, move the heading ‘controlled’ up

The formatting has been fixed as requested

15. Table 2, please clarify for the reader that you mean Texas wound grading. This needs to be clarified in both the legend and the table.

The Texas wound grading has been mentioned now in both the legend and the table as requested.

16. Figure 2, do you mean species instead of strain?

Yes, we mean species. We have subtitled it as requested
